# Relationship between blood lipid profiles and pancreatic islet β cell function in Chinese men and women with normal glucose tolerance: a cross-sectional study

**DOI:** 10.1186/1471-2458-12-634

**Published:** 2012-08-10

**Authors:** Tianpeng Zheng, Yun Gao, Haoming Tian

**Affiliations:** 1 Department of Endocrinology and Metabolism, West China Hospital of Sichuan University, Chengdu, 610041, People's Republic of China; 2 Department of Endocrinology, West China Hospital, Sichuan University, 37 GuoXue Street, Chengdu, Sichuan, 610041, China

**Keywords:** Lipids, Normal glucose tolerance, β cell function, Total cholesterol, Low-density lipoprotein–cholesterol

## Abstract

**Background:**

Dyslipidemia is present in people with diabetes as well as subjects with normal glucose tolerance (NGT). The purpose of this study was to investigate the relationship between lipid profiles and β cell function in Chinese individuals with NGT but without history of diabetes or prediabetes.

**Methods:**

A total of 893 men and 1454 women aged 18–76 years living in Sichuan, China, who were not being treated with lipid-lowering drugs were enrolled in this study. Insulin sensitivity (IR) was evaluated using the homeostasis model assessment –IR (HOMA-IR), β-cell function was calculated by the following equation: ΔI30/ΔG30/ HOMA-IR (ΔI30/ΔG30: the ratio of incremental glucose and insulin 30 min after glucose intake). Multivariate linear regression analyses were performed to estimate the relationship between blood lipid and β cell function as standardized coefficients (β).

**Results:**

β cell function decreased in men and women with increasing age. We found inverse relationships between β cell function and total cholesterol (TC) in men and women (β = −0.157 and −0.113, respectively, both p < 0.001), low-density lipoprotein–cholesterol (LDL-C; β = −0.130 and −0.068, respectively, both p < 0.001), TC/high-density lipoprotein–cholesterol (HDL-C) ratio (β = −0.084, p < 0.01 and −0.096, p < 0.001), and triglycerides (TG) (women only; β = −0.053, p < 0.05). However, β cell function was not associated with HDL-C in men or women (β = −0.034 and 0.000, respectively, both p > 0.05) or the TG/HDL-C ratio (β = −0.035 and −0.013, respectively, both p > 0.05). β cell function was significantly worse in males than in females in all age groups, except in subjects aged > 70 years.

**Conclusions:**

Dyslipidemia is associated with dysfunction of pancreatic β cells in subjects with NGT and this is particularly evident in people with elevated TC and LDL-C levels, especially males.

**Trial Registration Number:**

#TR-CCH-Chi CTR-CCH-00000361

## Background

Dyslipidemia, characterized by elevated low-density lipoprotein–cholesterol (LDL-C), triglyceride (TG), and total cholesterol (TC) levels, and lowered high-density lipoprotein–cholesterol (HDL-C) levels [1,2], is a major risk factor for cardiovascular disease. Some studies [3,4] have documented that dysfunction of pancreatic β cells caused by dyslipidemia precede the manifestation of type 2 diabetes mellitus (T2DM) and is an independent risk factor for the development of T2DM. Furthermore, the atherogenic lipid pattern is not only apparent in diabetic and prediabetic individuals but also in individuals with normal glucose tolerance (NGT). Although several studies have documented the impact of dyslipidemia on the function and survival of β cells in subjects with hyperglycemia, the relationship between β cell function and dyslipidemia in subjects with NGT remains to be clarified. Moreover, few studies have compared β cell function between men and women in different age groups, among subjects with dyslipidemia and NGT.

Our cross-sectional study, conducted between June and September 2007, was designed to provide reliable data on the relationship between β cell function and lipid profiles in Chinese individuals without prior history of diabetes or prediabetes.

In our study, the homeostasis model assessment of insulin resistance (HOMA-IR) was used to estimate insulin sensitivity. β-cell function was quantified as the ratio of the incremental insulin to glucose responses over the first 30 min during the Oral Glucose Tolerance Test (OGTT ) (ΔI30/ΔG30). Insulin sensitivity is known to be a critical modulator of the insulin response to a stimulus, with insulin resistance increasing insulin release. Thus, dividing ΔI30/ΔG30 by the HOMA-IR gave an adjusted measure of β-cell function (ΔI30/ΔG30/HOMA-IR) that accounted for variation in insulin sensitivity. This method has been used to assess β-cell function in previous large studies [5-8].

## Methods

### Subjects

This cross-sectional study was conducted as part of the China National Diabetes and Metabolic Disorders Study [9]. We used a multistage, stratified sampling method to select a representative sample of persons 18 years of age or older in the general population. The sampling process was stratified according to geographic region, degree of urbanization and economic development status. In the first stage of sampling, the whole Sichuan province was stratified into urban and rural areas. Two sample points from urban areas and three from rural areas were selected to be representative of the geographic and economic characteristics in their regions. In the second stage, 2 street districts or rural villages were randomly selected from each sample point, respectively. Primary sampling units were street districts in urban areas and hamlets in rural sample points. In the third stage, one participant was selected from a randomly selected household in the primary sampling units. Simple random sampling methods were used at each stage. A total of 3514 subjects, including 1400 men and 2114 women, aged 18–76 years, participated in this survey between June and September 2007. Of the 3514 participants, 2347 with NGT [fasting plasma glucose (FPG) < 109.8 mg/dl, and 2-h post oral glucose tolerance test plasma glucose < 140.4 mg/dl] [10], and complete data were included in this study. Subjects with diabetes or prediabetes [including isolated impaired fasting glucose (IFG), isolated impaired glucose tolerance (IGT), or IFG plus IGT] were excluded. After enrollment, subjects were stratified by sex. There were 2347 subjects ( 893 males and 1454 females),out of which there were 349 postmenopausal women. At present 45 subjects were on diuretics, 78 were on calcium channel blocker (CCB) and 53 others were on Angiotensin-Converting Enzyme Inhibitors (ACEI) or Angiotensin Receptor Blocker (ARB) therapy.

The study was approved by the Drugs/Medical Apparatus & Instruments Ethics Committee at China Japan Friendship Hospital (07020470055), and all subjects gave their informed consent. This study was registered on the Chinese clinical trial registry (#TR-CCH-Chi CTR-CCH-00000361).

### Study design

A standard questionnaire was administered by trained staff to participants to record demographic characteristics and lifestyle risk factors [11]. Blood pressure, body weight, height, waist and hip circumference, body mass index (BMI), and waist/hip ratio (WHR) were measured and calculated using standard methods, as previously described [9]. Participants were instructed to maintain their usual physical activity and diet for at least 3 days before undergoing an OGTT. After an overnight fast ≥ 10 h, a venous blood specimen was collected to measure FPG, fasting insulin and blood lipids (including TC, TG, LDL-C, and HDL-C). Blood samples were also drawn at 30 and 120 min after a 75 g glucose load to measure glucose and insulin concentrations. The blood samples were placed in an ice box and transported to the laboratory of endocrinology and metabolism at West China Hospital, Sichuan University, for analysis. Plasma glucose levels were measured using a hexokinase enzymatic method. Insulin was measured by a radioimmunoassay with human insulin as a standard (Linco, St Charles, MO). TG, TC, LDL-C, and HDL-C levels were determined enzymatically. β cell function was calculated from insulin and glucose levels determined at 0 and 30 min using the following equation: (ΔI30 / ΔG30) / HOMA-IR, where the incremental glucose (ΔG30) and insulin (ΔI30) responses were calculated as the difference in values between 30 and 0 min. The homeostasis model assessment of insulin resistance (HOMA-IR) was calculated from FPG and fasting insulin levels as described by Matthews et al. [12] using the equation: (FPG × fasting insulin) / 22.5.

### Statistical analysis

All analyses were performed using SPSS software version 16.0 (SPSS Inc., Chicago, IL). Since the distributions of fasting insulin, 0.5-h insulin, 2-h insulin, and TG were skewed, these parameters were logarithmically transformed before statistical analysis. Clinical and biochemical characteristics were compared using Student’s *t* test or *χ*^2^ tests. Sex-specific mean values of β cell function were calculated for 10 mg/dl cut-off levels of TC (< 120, 120–139.9, 140–159.9, 160–179.9, 180–199.9, 200–219.9, and >220 mg/dl; n = 47, 104, 173, 194, 158, 124, and 93, respectively, for men; n = 65, 147, 319, 328, 264, 172, and 159, respectively, for women), for 25 mg/dl cut-off levels of TG (< 74.9, 75–99.9, 100–124.9, 125–149.9, 150–174.9, 175–199.9, and > 200 mg/dl; n = 165, 193, 157, 103, 82, 68, and 125, respectively, for men; n = 364, 375, 260, 156, 106, 52, and 141, respectively, for women), for 10 mg/dl cut-off levels of HDL-C (< 29.9, 30–39.9, 40–49.9, 50–59.9, 60–69.9, and > 70 mg/dl; n = 29, 201, 335, 205, 88, and 35, respectively, for men; n = 30, 204, 405, 444, 247, and 124, respectively, for women), for 15 mg/dl cut-off levels for LDL-C (< 84.9, 85–99.9, 100–114.9, 115–129.9, 130–144.9, 145–159.9, and > 160 mg/dl; n = 192, 166, 150, 132, 95, 74, and 84, for men; n = 360, 315, 281, 220, 110, 94, and 74, respectively, for women), for 0.5 unit cut-off levels of TC/HDL ratio (< 2.49, 2.5–2.99, 3.0–3.49, 3.5–3.99, 4.0–4.49, 4.5–4.99, and >5.0; n = 59, 138, 191, 180, 125, 90, and 110, respectively, for men; n = 167, 316, 357, 241, 150, 111,and 112, respectively, for women), for 1 unit cut-off levels of TG/HDL ratio (< 1.49, 1.5–2.49, 2.5–3.49, 3.5–4.49, 4.5–5.49, and > 5.5; n = 197, 262, 172, 102, 49, and 111, respectively, for men; n = 482, 489, 196, 136, 65, and 86, respectively, for women), and for 10-year age groups (< 30, 30–39, 40–49, 50–59, 60–69, and >70 years; n = 193, 229, 145, 151, 145, and 30, respectively, for men; n = 292, 355, 303, 265, 191, and 48, respectively, for women). Multiple linear regression analyses were used to examine the relationship between β cell function as the dependent variable and blood lipid levels as independent variables. Standardized coefficients (β) were determined after adjusting for age, family history of diabetes, level of activity, BMI, smoking, and systolic blood pressure (SBP). Multiple linear regression was also used to examine the relationship between β cell function and age, adjusting for family history of diabetes, level of activity, BMI, smoking, and SBP. T tests were used to compare sex-specific β cell function among subjects in different age groups.

## Results

### Clinical and biochemical characteristics

Table 1 shows the sex-specific clinical and biochemical characteristics. There were no differences in age, family history of diabetes, regular leisure-time physical activity, BMI, WC, SBP, FPG, 0.5-h plasma glucose, 2-h plasma glucose, fasting insulin, TC, HDL-C, or LDL-C between males and females. However, compared with women, more men were smokers or consumed alcohol, they more frequently had college education and higher income, and they had higher WHR. Men also had lower 0.5-h insulin, 2-h insulin, and TG levels.

**Table 1:** Subject characteristics*

**Characteristic**	**Men (n = 893)**	**Women (n = 1454)**	**p value**
Age (years)	42.6 ± 14.9	43.3 ± 14.2	NS
Family history of diabetes (%)	11.9	12.3	NS
Cigarette smoking (%)	49.7	4.3	< 0.001
Alcohol drinking (%)	55.5	11.5	< 0.001
Education			
College or above (%)	32.6	25.0	< 0.01
Middle school (%)	39.1	40.1	NS
Primary school or below (%)	28.3	34.9	< 0.01
Income (per year)			
≥10000 yuan RMB (%)	32.7	27.6	< 0.01
<10000 yuan RMB (%)	67.3	72.4	< 0.01
Regular leisure-time physical activity (%)	58.5	62.6	NS
BMI (kg/m^2^)	22.7 ± 3.2	22.3 ± 3.4	NS
WC (cm)	81.1 ± 9.7	78.1 ± 9.8	NS
WHR	0.88 ± 0.08	0.82 ± 0.08	0.019
SBP (mmHg)	115.7 ± 16.5	111.3 ± 17.7	NS
fasting plasma glucose (mg/dl)	83.5 ± 9.6	83.2 ± 9.1	NS
0.5-h plasma glucose (mg/dl)	143.4 ± 30.4	135.7 ± 28.1	NS
2-h plasma glucose (mg/dl)	96.2 ± 21.6	99.3 ± 19.1	NS
Fasting insulin(μU/ml)	6.3 (4.4, 8.8)	6.6 (4.7, 9.3)	NS
0.5-h insulin (μU/ml)	28.4 (13.2, 42.1)	33.2 (13.0, 48.2)	< 0.01
2-h insulin (μU/ml)	15.2 (8.0, 28.8)	19.4 (11.3, 37.1)	0.023
TC (mg/dl)	173.9 ± 35	176.1 ± 38.2	NS
TG (mg/dl)	98.2 (74.3, 136.3)	112.4 (81.4, 165.5)	0.01
HDL-C (mg/dl)	48.2 ± 16.4	52.5 ± 13.9	NS
LDL-C (mg/dl)	106.3 ± 30.9	105.1 ± 31.9	NS

Data were expressed as means ± standard deviation, median (interquartile range), or percentage for normally distributed continuous various, abnormally distributed continuous variables, and categorical variables, respectively. Abnormally distributed variables were logarithmically transformed before analysis. Between-group comparisons were conducted using Student’s *t* test or *χ*^2^ tests. NS: not significant; BMI: body mass index; WC: waist circumference; WHR: waist/hip ratio; SBP: systolic blood pressure; TG: triglyceride; TC: total cholesterol; HDL-C: high-density lipoprotein–cholesterol; LDL-C: low-density lipoprotein–cholesterol. Cigarette smoking was defined as having smoked at least 100 cigarettes in one’s lifetime. Alcohol consumption was defined as consumption of ≥ 30 g of alcohol per week for 1 year or more. Regular leisure-time physical activity was defined as participation in ≥ 30 min of moderate or vigorous activity per day at least 3 days per week.

### Relationship between β cell function and blood lipid profiles

**Figure 1:**
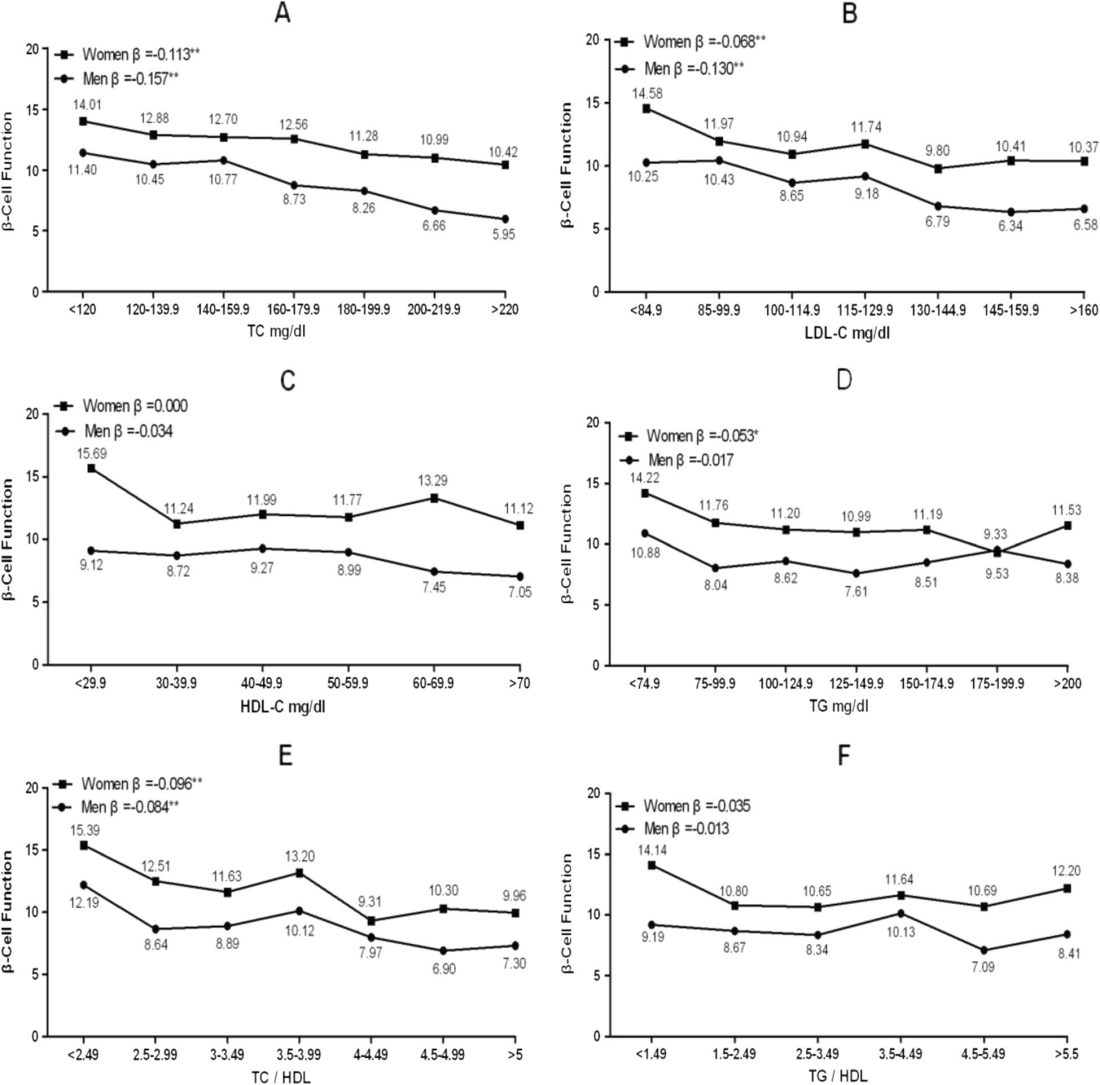
**Associations between β cell function and blood lipid parameters in men and women with normal glucose tolerance. **Standardized coefficients (β) were estimated after adjusting for age, body mass index, smoking, and systolic blood pressure, leisure-time physical activity, family history of diabetes. *p < 0.05 and **p < 0.01.

Multivariate linear regression analyses (Figure 1, Table 2) showed inverse trends between β cell function and TC in both men and women [β = −0.157 (corresponding to a change in β cell function of 0.157 units for each 1-mg/dl increase in FPG) and −0.113, respectively, both p < 0.001], LDL-C (β = −0.130 and −0.068, respectively, both p < 0.001), TC/HDL ratio (β = −0.084 and −0.096, respectively, both p < 0.01), and TG in women (β = −0.053, p < 0.05) after adjustment for potential confounding factors. On the other hand, β-cell function was not associated with HDL-C in men or women (β = −0.034 and −0.000, respectively, both p > 0.05) or TG/HDL-C ratio (β = −0.034 and −0.000, respectively, both p > 0.05).

**Table 2:** Multiple linear regression analyses of variables associated with β cell function in NGT subjects

	**Male**		**Female**		**Male**		**Female**		**Male**		**Female**		**Male**		**Female**		**Male**		**Female**		**Male**		**Female**	
**variables**	β	P	β	P	β	P	β	P	β	P	β	P	β	P	β	P	β	P	β	P	β	P	β	P
**TC (mg/dl)**	**−0.157**	**<0.001**	**−0.113**	**<0.001**	**-**	**-**	**-**	**-**	**-**	**-**	**-**	**-**	**-**	**-**	**-**	**-**	**-**	**-**	**-**	**-**	**-**	**-**	**-**	**-**
**TG (mg/dl)**	**-**	**-**	**-**	**-**	**−0.017**	**0.742**	**−0.053**	**0.045**	**-**	**-**	**-**	**-**	**-**	**-**	**-**	**-**	**-**	**-**	**-**	**-**	**-**	**-**	**-**	**-**
**HDL-C (mg/dl)**	**-**	**-**	**-**	**-**	**-**	**-**	**-**	**-**	**−0.034**	**0.345**	**0.000**	**0.885**	**-**	**-**	**-**	**-**	**-**	**-**	**-**	**-**	**-**	**-**	**-**	**-**
**LDL-C (mg/dl)**	**-**	**-**	**-**	**-**	**-**	**-**	**-**	**-**	**-**	**-**	**-**	**-**	**−0.130**	**<0.001**	**−0.068**	**<0.001**	**-**	**-**	**-**	**-**	**-**	**-**	**-**	**-**
**TC/HDL-C**	**-**	**-**	**-**	**-**	**-**	**-**	**-**	**-**	**-**	**-**	**-**	**-**	**-**	**-**	**-**	**-**	**−0.084**	**<0.01**	**−0.096**	**<0.001**	**-**	**-**	**-**	**-**
**TG/HDL-C**	**-**	**-**	**-**	**-**	**-**	**-**	**-**	**-**	**-**	**-**	**-**	**-**	**-**	**-**	**-**	**-**	**-**	**-**	**-**	**-**	**−0.013**	**0.698**	**−0.035**	**0.181**
**Age(year)**	**−0.115**	**<0.01**	**−0.078**	**0.010**	**−0.135**	**<0.001**	**−0.092**	**<0.01**	**−0.135**	**<0.01**	**−0.095**	**<0.01**	**−0.125**	**<0.001**	**−0.090**	**<0.01**	**−0.124**	**<0.001**	**−0.077**	**0.01**	**−0.134**	**<0.001**	**−0.094**	**<0.01**
**BMI (kg/m**^**2**^**)**	**0.098**	**<0.01**	**−0.039**	**0.194**	**0.062**	**0.098**	**0.041**	**0.176**	**0.061**	**0.088**	**−0.043**	**0.150**	**0.081**	**0.024**	**−0.042**	**0.164**	**0.084**	**0.021**	**−0.036**	**0.230**	**0.065**	**0.077**	**−0.042**	**0.158**
**SBP (mmHg)**	**−0.055**	**0.133**	**−0.041**	**0.174**	**−0.072**	**0.052**	**−0.049**	**0.098**	**−0.070**	**0.057**	**−0.051**	**0.089**	**−0.067**	**0.067**	**−0.048**	**0.106**	**−0.069**	**0.060**	**−0.052**	**0.080**	**−0.072**	**0.052**	**−0.050**	**0.095**
**Smoking**	**−0.253**	**<0.001**	**−0.048**	**0.089**	**−0.265**	**<0.001**	**−0.049**	**0.084**	**−0.261**	**<0.001**	**−0.048**	**0.086**	**−0.258**	**<0.001**	**−0.048**	**0.085**	**−0.272**	**<0.001**	**−0.046**	**0.104**	**−0.265**	**<0.001**	**−0.049**	**0.084**
**Leisure-time** **physical activity**	**0.169**	**<0.001**	**0.045**	**0.092**	**0.162**	**<0.01**	**0.055**	**0.037**	**0.161**	**<0.01**	**0.056**	**0.036**	**0.173**	**<0.001**	**0.052**	**0.051**	**0.166**	**<0.001**	**0.058**	**0.028**	**0.162**	**<0.01**	**0.055**	**0.039**
**Family history** **of diabetes**	**0.004**	**0.915**	**0.008**	**0.755**	**0.003**	**0.925**	**0.010**	**0.713**	**0.007**	**0.836**	**0.010**	**0.711**	**0.005**	**0.891**	**0.009**	**0.726**	**−0.004**	**0.901**	**0.012**	**0.647**	**0.004**	**0.897**	**0.010**	**0.718**

### Sex-specific β cell function according to age

**Figure 2:**
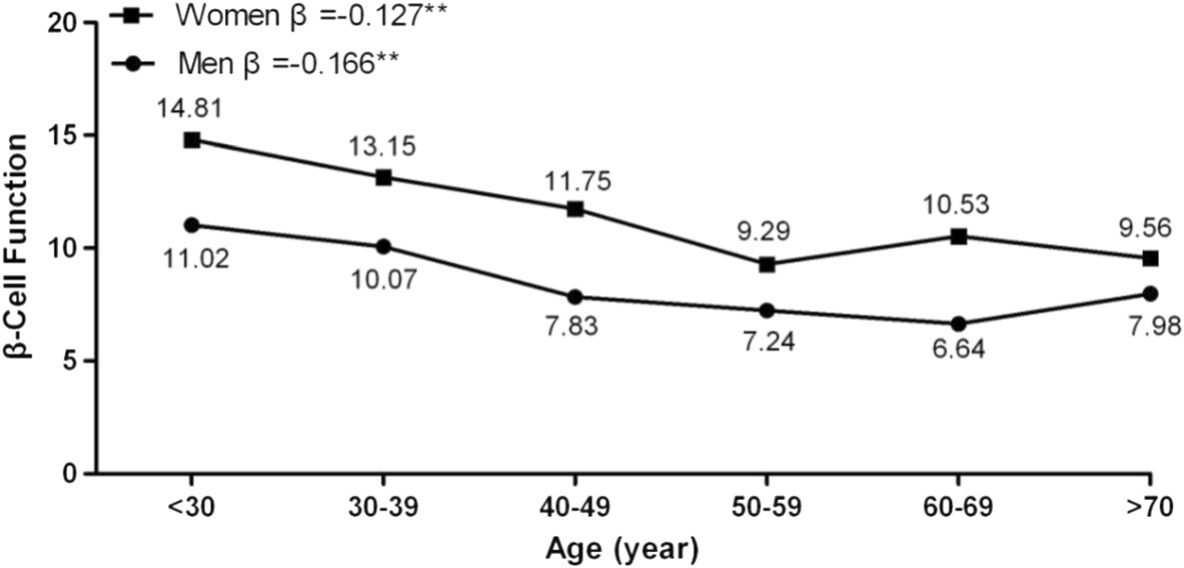
**Association between β cell function and age in men and women with normal glucose tolerance. **Standardized coefficients (β) were estimated after adjusting for family history of diabetes, level of activity, BMI, smoking, and SBP. *p < 0.05 and **p < 0.01.

Figure 2 shows sex-specific β cell function according to age after adjustment for family history of diabetes, level of activity, BMI, smoking, and SBP. This analysis showed an inverse trend between β cell function and age in both men and women (β = −0.166 and −0.127, respectively, both p < 0.001). Table 3 shows that β cell function was significantly worse in men than in women in all age groups, except in subjects aged > 70 years.

**Table 3:** Comparison of β cell function between men and women according to age

**Age (years)**	**n (male:female)**	**β cell function**		**p value**
		**Men**	**Women**	
< 30	485 (193:292)	11.0 ± 5.8	14.8 ± 7.2	< 0.001
30-39	584 (229:355)	10.1 ± 4.9	13.2 ± 5.8	< 0.01
40-49	448 (145:303)	7.8 ± 3.9	11.8 ± 4.3	< 0.001
50-59	416 (151:265)	7.2 ± 3.7	9.3 ± 4.1	0.01
60-69	336 (145:191)	6.6 ± 2.8	10.5 ± 3.8	< 0.01
> 70	78 (30:48)	8.0 ± 3.1	9.6 ± 4.2	NS

Data are expressed as means ± standard deviation. Between-group comparisons were conducted using Student’s *t* test.

## Discussion

It is well established that cholesterol homeostasis is fundamental for appropriate insulin secretory function of β cells [13,14], as excessive cholesterol accumulation in β cells may cause lipotoxicity and reduce insulin secretion, causing β cell dysfunction and decreased β cell mass [15-17]. Some studies [18] have documented that people with diabetes or prediabetes had higher TC levels when compared with individuals with NGT. This might contribute to the progression from the insulin-resistant state to overt T2DM, and subsequent deterioration of T2DM. In our study, we found that β cell was inversely associated with TC and LDL-C in men and women with NGT, and that β cell function deteriorated with increasing level of TC and LDL-C. On one hand, the reason may be due to the deterioration of the pancreatic β cell function aggravated by disorder in lipid metabolism, the mechanisms for this change could be summarized as follows. First, the accumulation of cholesterol in β cells decreases the expression of the transcription factors pancreatic and duodenal homeobox 1 and β cell E-box trans-activator 2, both of which are important in β cell development and survival [19]. Second, high β cell cholesterol levels interfere with glucose metabolism by reducing the effectiveness of the insulin secretory apparatus and inhibiting glucokinase activity [14]. Third, increased cholesterol levels in β cells may lead to deficient ATP-binding cassette transporter A1 (ABCA1) activity, which promotes cholesterol efflux from cells to lipid-free/lipid-poor apolipoprotein A-I in the extracellular space [13]. Recent studies [20] have also demonstrated that LDL-C inhibits glucose-stimulated insulin secretion and β cell proliferation through LDL-receptor dependent and independent mechanisms, respectively. Furthermore, Roehrich et al. [21] reported that high LDL-C levels (*>* 6 mmol/l) induce apoptosis of β cells. On the other hand, as our study is a cross-sectional study, the results may also be due to impaired pancreatic β cell functions which in turn lead to lipid metabolism disorders. Insulin resistance is mainly found in early stages of impaired islet β-cell function. Previous studies have confirmed that under the presence of insulin resistance, suppression of FFA by insulin is reduced and the increased level of FFA will enhance small dense LDL level whereas the overall level of LDL-C will be less affected [22]. Our data appear to provide further evidence that elevated levels of TC and LDL-C is associated with β cell dysfunction in subjects with NGT, as well as in people with diabetes or prediabetes. But we still need more prospective studies to clarify the relationship between pancreatic islet β-cell dysfunction and disorders in lipid metabolism.

Several *in vivo* and *in vitro* studies [23,24] have shown that hypertriglyceridemia diminishes glucose-induced insulin secretion via the glucose–fatty acid cycle in which fatty acid oxidation inhibits glucose oxidation by decreasing pyruvate dehydrogenase (PDH) activity and increasing PDH kinase activity. Moreover, elevated TG levels induce β cell apoptosis by increasing the levels of ceramide and nitric oxide [25,26]. Islet β-cell dysfunction can also lead to elevated TG levels, the mechanism could be that under the insulin resistance condition, decrease in LPL activity and excessive increase in VLDL level will eventually reduce the decomposition in chylomicrons (CM) [27]. However, the association between β cell function and TG in our study was weaker than the associations of β cell function with TC or LDL-C, and the association was only apparent in women. There are several possible reasons for this discrepancy. First, TG levels fluctuate widely depending on recent dietary intake or weight changes; some of the enrolled subjects may not have followed our instructions strictly and consumed foods rich in TG before blood sampling. Second, there may be sex difference in TG levels meaning further studies are needed. Third, compared with TC and LDL-C, TG may exert weaker effects on β cell function in subjects with NGT.

Regarding HDL-C, we did not find any evidence for a relationship between HDL-C and β cell function. Although previous studies have shown that HDL-C stimulates insulin secretion by interacting with ABCA1, the ATP-binding cassette transporter G1, or scavenger receptor B1, it also inhibits apoptosis of β cells [4]. Other pre-diabetes and diabetes population studies also showed that the pancreatic β cell dysfunction can lead to elevation in HDL-C levels, and the mechanism may be associated with alteration in VLDL catabolism, decrease in apolipoprotein AI (ApoAI) levels and insufficient LPL functions. At present time few research has been done on the the NGT group regarding the relationship between pancreatic β cell function and HDL-C, and the results of our study are inconsistent with previous researches done in the impaired glucose tolerance groups. The specific reason for this inconsistency is still unknown, it was estimated that HDL-C may play an important role in protecting β cells in relatively late stages of disease, particularly in IFG, IGT, or T2DM, while its protective effects in NGT are less apparent. Further studies are needed to examine the relationship between β cell function and HDL-C in subjects with NGT, IFG, IGT, and T2DM.

Our data indicate that β cell function is impaired not only in people with IFG, IGT, or T2DM, but also in individuals with NGT and dyslipidemia, particularly in subjects with elevated levels of TC and LDL-C. Compared with TG and HDL-C, TC and LDL-C have a more significant relationship with pancreatic β cell functions at a relatively early stage. Consequently, high levels of TC and LDL-C in NGT subjects with impaired β cell function should be paid more attention. We believe that exercise and diet therapy should be preferred to these people first because these two modalities of treatment have clear effect in alleviating insulin resistance and blood lipids metabolism disorder. Whether the incidence of developing diabetes or atherosclerosis is higher in these group than normal individuals and whether the treatment for these people using lipid lowering agents or drugs increasing insulin sensitivity have not been clear yet, we still need more prospective studies to make further confirmation.

The TC/HDL-C and TG/HDL-C ratios are both recognized as effective predictors of cardiovascular disease risk [28,29]. Similarly, Giannini et al. [30] reported that the TG/HDL-C ratio is associated with insulin resistance and may be used with other risk factors to identify subjects at increased risk of insulin resistance-driven morbidity. In assessment of β cell function, however, our analysis revealed that the TC/HDL-C ratio, but not TG/HDL-C ratio, increased with decreasing β cell function. Reasons for this difference may be include differences in diet, sex, or other factors.

Our finding that β-cell function decreased in men and women with increasing age is consistent with the results of other studies [18,31]. Interestingly, we also found that β cell function was significantly worse in males than in females in each age group, except in subjects aged > 70 years. Different levels of blood lipids in males and females in each age group may partly explain these sex differences. Previous studies have already reported different changes in blood lipids between men and women with increasing age. For example, the Lipid Research Clinics Program Study [31], a cross-sectional study of a North American Caucasian population, showed that TC increased with age up to the age of 50–54 years in men but declined with age up to 55–59 years in women. By contrast, both TC and TG increased with age up to the oldest age group of 55–59 years in both sexes. The trends in blood lipid levels in men and women with age in Chinese individuals are still not fully understood and may differ from those in western populations. Clearly, prospective studies in China are needed to further investigate these trends. Notably, however, Yang et al. [9] reported that the prevalence rates of diabetes (10.6% vs. 8.8%) and prediabetes (16.1% vs. 14.9%) were higher in men than women. These results indicate that males with NGT and dyslipidemia should be paid more attention than females.

Our study has several limitations. First, β cell function did not differ between males and females > 70 years of age. This may due to the relatively small number of subjects in this age group, and could be overcome by increasing the number of subjects in this age group. Second, because of the cross-sectional design, we cannot further clarify the mutual interaction between the disorder in lipid metabolism and pancreatic β cell dysfunction.

## Conclusions

Dyslipidemia is associated with dysfunction of pancreatic β cells in subjects with NGT and this is particularly evident in people with elevated TC and LDL-C levels, especially males.

## Competing interest

The authors declare that they have no conflict of interest.

## Authors’ contributions

TPZ performed the statistical analysis and prepared the manuscript. HMT was responsible for study design and coordination, guided the statistical analysis, and revised the manuscript. YG was responsible for study design and coordination, and critically reviewed the manuscript. TPZ and YG performed data collection and reviewed the manuscript. All authors read and approved the final manuscript.

## Pre-publication history

The pre-publication history for this paper can be accessed here:

http://www.biomedcentral.com/1471-2458/12/634/prepub
